# Ethnobotanical survey and scientific validation of liver-healing plants in northeastern Morocco

**DOI:** 10.3389/fphar.2024.1414190

**Published:** 2024-09-10

**Authors:** Noureddine Bencheikh, Amine Elbouzidi, Abdellah Baraich, Mohamed Bouhrim, Abdelhamid Azeroual, Mohamed Addi, Ramzi A. Mothana, Hanan M. Al-Yousef, Bruno Eto, Mostafa Elachouri

**Affiliations:** ^1^ Agri-Food and Health Laboratory (AFHL), École Supérieure Normale, Hassan First University, Settat, Morocco; ^2^ Laboratoire d’Amélioration des Productions Agricoles, Biotechnologie et Environnement (LAPABE), Faculté des Sciences, Université Mohammed Premier, Oujda, Morocco; ^3^ Laboratory of Bioressources, Biotechnology, Ethnopharmacology and Health, Faculty of Sciences, Mohammed First University, Oujda, Morocco; ^4^ Laboratory of Biological Engineering, Team of Functional and Pathological Biology, University Sultan Moulay Slimane Faculty of Sciences and Technology Beni Mellal, Meknes, Morocco; ^5^ Department of Pharmacognosy, College of Pharmacy, King Saud University, Riyadh, Saudi Arabia; ^6^ Laboratories TBC, Laboratory of Pharmacology, Pharmacokinetics and Clinical Pharmacy, Faculty of Pharmacy, University of Lille, Lille, France

**Keywords:** ethnobotany, ethnopharmacology, traditional medicine, medicinal plants, liver diseases

## Abstract

**Introduction:**

Liver diseases represent a significant global health challenge, with primary causes including excessive alcohol consumption, infections, chemotherapy, and autoimmune disorders. Medicinal plants, due to their natural bioactive compounds, hold promise for developing effective treatments and preventive measures against liver ailments. This study aimed to document the use of herbal remedies in northeastern Morocco for liver diseases and correlate these uses with scientific evidence through a bibliometric analysis.

**Methods:**

An ethnobotanical survey was conducted in remote communities of northeastern Morocco from October 2020 to January 2022. A total of 189 informants were interviewed using semi-structured questionnaires to gather information on local medicinal plants used for liver ailments. The data were analyzed using four ethnobotanical quantitative indices: use value (UV), familial use value (FUV), informant consensus factor (ICF), and fidelity level (FL). Additionally, a bibliometric analysis was performed to evaluate the scientific support for the ethnopharmacological uses documented.

**Results:**

The survey identified 45 plant species from 26 different families used in the treatment of liver diseases. The most frequently utilized species were *Cuminum cyminum* L. (UV = 0.1065), *Allium sativum* L. (UV = 0.1015), *Salvia officinalis* L. (UV = 0.0761), *Asparagus officinalis* L. (UV = 0.0558), and *Ziziphus lotus* (L.) Lam. (UV = 0.0457). The Apiaceae family showed the highest familial use value (FUV = 0.1066), followed by Alliaceae (FUV = 0.1015). Liver congestion had the highest informant consensus factor (ICF = 0.83), followed by hepatic colic (ICF = 0.80). Bibliometric analysis revealed that 61% of the plants identified had documented pharmacological effects related to liver health.

**Discussion:**

The study demonstrates that traditional knowledge in northeastern Morocco encompasses a rich diversity of medicinal plants used to treat liver diseases. The high ICF values indicate a strong consensus among informants on the efficacy of these remedies. The correlation between ethnopharmacological use and scientific validation for a significant portion of these plants suggests their potential as reliable therapeutic agents for liver conditions. However, further scientific investigations are necessary to confirm their efficacy and safety in clinical settings. This research contributes valuable information for future studies on the therapeutic potential of these plants.

**Conclusion:**

This ethnobotanical survey provides a comprehensive database of medicinal plants used in northeastern Morocco for liver diseases. The findings highlight the potential of these plants in developing novel treatments for hepatic conditions, although further research is essential to substantiate their therapeutic claims.

## 1 Introduction

The liver is one of the most critical organs in the human body, playing a pivotal role in several physiological functions, including the regulation of metabolic processes, maintenance of blood sugar levels, bile production, and detoxification of foods, water, drugs, and xenobiotics (Marcellin and Kutala, 2018). These functions are vital for sustaining overall health, as the liver processes everything that enters the body, ensuring that nutrients are metabolized correctly and harmful substances are neutralized. Given its extensive involvement in maintaining homeostasis, the liver is susceptible to various diseases, which can manifest as serious clinical syndromes such as jaundice, hepatitis, hepatocarcinoma, and cirrhosis. Because of its essential functions, the liver is often considered a reflection of an individual’s overall health (Rinder et al., 2011).

Liver dysfunction is a significant global health problem, with a variety of causes that contribute to its widespread prevalence. These causes include excessive alcohol consumption, infections (notably viral hepatitis), the use of chemotherapeutic agents, exposure to toxic chemicals, and autoimmune disorders (Wei et al., 2022). The impact of liver diseases is profound, with global mortality rates reaching approximately 2 million deaths annually. Of these, 1 million deaths are attributed to complications arising from viral hepatitis and hepatocellular carcinoma, while another 1 million result from cirrhosis (Asrani et al., 2019). The growing burden of liver diseases has underscored the urgent need for effective therapeutic strategies, particularly in regions where access to conventional medical treatments is limited.

Medicinal plants have long been recognized as a valuable source of therapeutic agents, offering potential remedies for a wide array of health conditions, including liver diseases (Bhagawan et al., 2023a). The use of plants in traditional medicine is deeply rooted in human history, with ethnobotanical practices providing insights into natural remedies that have been utilized for centuries (Bhagawan et al., 2022; 2023b; 2024). In Morocco, traditional herbal medicine remains a cornerstone of healthcare, especially in rural and underserved areas. Recent ethnobotanical research indicates that a significant proportion of the Moroccan population—ranging from 60% to 80%—relies on medicinal plants to meet their healthcare needs (Jamila and Mostafa, 2014; Labiad et al., 2020; Alami Merrouni et al., 2021; Fakchich and Elachouri, 2021; Bencheikh et al., 2022; 2023). This reliance is driven by several factors, including the high cost of conventional medications, limited access to adequate healthcare facilities, and socio-economic challenges, particularly in remote and underdeveloped regions (Bencheikh et al., 2021e; Fakchich and Elachouri, 2021).

The cultural heritage of North-Eastern Morocco, like that of other regions in the country, is steeped in a rich tradition of herbal medicine that dates back to the Arab influence in the 7th century. Over centuries, the indigenous population has developed and maintained extensive knowledge of medicinal plants, which forms the foundation of the region’s traditional medical system. This knowledge is passed down orally from one generation to the next, ensuring the continuity of these traditional practices. However, this oral transmission is also a source of vulnerability. The absence of formal documentation and the lack of ethnobotanical archives pose significant threats to the preservation of this cultural heritage. As modern influences encroach and younger generations turn to contemporary medicine, there is a real risk that this indigenous medicinal knowledge, along with the phytogenetic resources it depends on, could be lost (Eddouks et al., 2017).

In Morocco, despite the widespread use of traditional medicine, there is a notable gap in ethnobotanical documentation, particularly concerning medicinal plants used for treating liver diseases. This lack of documented evidence limits the potential for scientific validation and integration of these traditional practices into modern healthcare systems. To address this gap, we propose a study aimed at documenting and analyzing the traditional knowledge related to medicinal plants used in rural areas of North-East Morocco for the treatment of liver diseases. The study will also seek to correlate these traditional uses with scientific evidence through a bibliometric review, thereby providing a comprehensive understanding of the therapeutic potential of these plants and contributing to the preservation of Morocco’s ethnobotanical heritage.

## 2 Materials and methods

### 2.1 Study area

**FIGURE 1:**
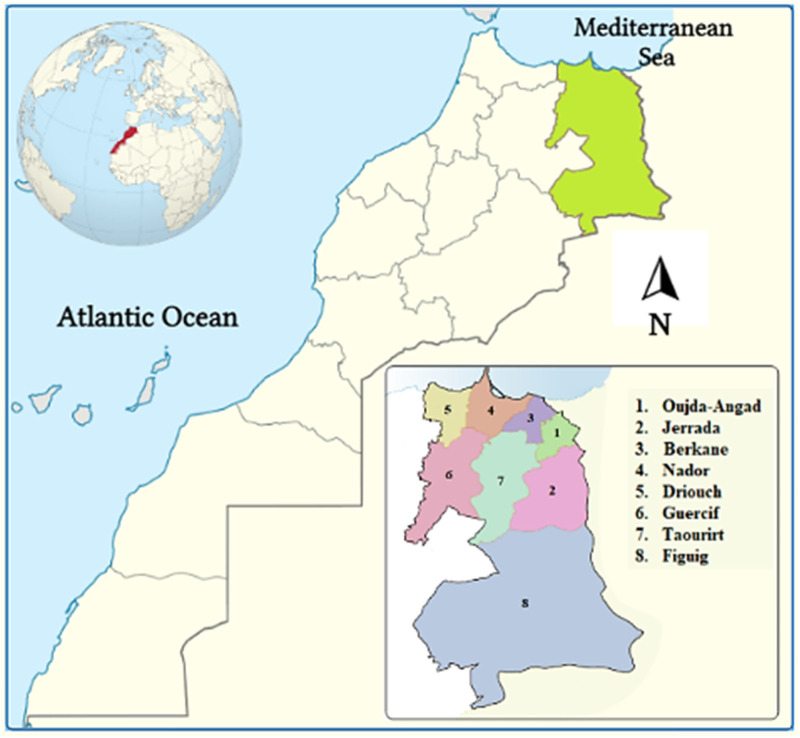
Geographic location of the study area.

The Eastern region of Morocco, covers 90,130 km^2^, or 12% of the country’s total size (Figure 1). This region is limited to the West by the provinces of Al Hoceima, Taza, Boulmane, and Errachidia, to the North by the Mediterranean, to the East, and to the South by the Morocco-Algerian border. The population of this region reached 2,314,346 people (6.8% of the total population), with a density of 26 people per square kilometer, according to the national census report issued in 2014 (RGPH, 2014). According to the High Commission for Planning’s survey, the dialect of Arabic was spoken here the most frequently, followed by Berber or Tamazight, which is split into two tiny dialects: Tarifit in the north and tachelhit in the south. The territory’s southern zone is characterized by the vast Highlands and Sahara, while the mountainous areas of Beni Snassen, Rif, and Horst reach 1800 m, 1,500 m, and 1,100 m, respectively, elevations. The region also has 200 km of Mediterranean coastline. With hot, dry summers and cooler, humid winters, the region has a Mediterranean climate zone, with average annual rainfall ranging from 100 mm in the south to 400 mm in the north. Additionally, the area has a number of protected areas and sites of biological and ecological interest, including Al Hoceima National Park, Benisnassen, Jbel Gorougou, Cap des Trois Fourches, Chekhar, Lalla Chafia, and Lalla Mimouna. In fact, these places had already been chosen because of their biological and ecological characteristics as well as their indigenous flora (Fennane, 2004; Fakchich and Elachouri, 2021).

### 2.2 Ethnobotanical data collection

The collection of ethnobotanical data on liver diseases was conducted between October 2020 and January 2022 across twelve rural communes located in five provinces of northeastern Morocco. Traditional knowledge was randomly selected from twelve stations studied through structured and semi-structured interviews using a questionnaire sheet with 189 local residents and 8 traditional herbalists participated. Verbal informed consent was gained from informants following verbal explanation of the study aims. The established best practice for ethnobotanical investigations, the International Society of Ethnobiology’s Code of Ethics, was followed when conducting interviews (International Society of Ethnobiology, 2006). The questionnaire sheet utilized in this study has two sections: the first lists the respondents’ demographic information, and the second lists their floristic and ethnic backgrounds.

### 2.3 Identification of specimens

We were able to transform the common names of plants identified during our ethnobotanical survey into their botanical names using some relevant references (Bellakhdar et al., 1991; Jamila and Mostafa, 2014). Subsequently, plant samples were collected from various vegetation sites across the northeastern region of Morocco. After the harvest, the botanical identification of the samples was carried out in the Laboratory of Bioresources, Biotechnologies, Ethnopharmacology and Health of the Faculty of Sciences of Mohammed first University, Oujda, Morocco, with the help of available herbaria and a number of essential references such as the catalogue of Moroccan plants and the practical flora of Morocco (Jahandiez and Maire, 1931; 1932; 1934; Fennane et al., 1999; 2007; 2014). After the samples were identified, specimens were placed in the Mohammed First University Herbarium in Oujda, Morocco. Using the World Flora Online (WFO) Plants database (https://wfoplantlist.org/), all scientific names were reviewed once more. Additionally, a group of flowering plants (angiosperms) known as Angiosperm Phylogeny Group III - 2009 has been given credit for naming all plant families (A.P.G III, 2009).

### 2.4 Quantitative data analysis

To quantify the ethnobotanical information, we adopted a quantitative analysis using ethnobotanical indices such as the Medicinal Use Value (UV), the Family Use Value (FUV), Informant Consensus Factor (ICF), and the Fidelity Level (FL).

• Medicinal use value (UV)

We analyzed the medicinal use value of each plant species to identify the relative relevance of each plant species that is locally recognized to be utilized in herbal treatments. This index is calculated using the formula below (Tabuti et al., 2003):

$$\text{UV}=\frac{\sum U}{N}$$

Where;

**UV**: medicinal use value, **U:** number of citations per species, **N:** number of informants. The UV value will be larger if a plant has a high utilization ratio, indicating that the plant is significant, however if there are few utilization ratios, it will be near to zero.

• Botanical Family Use Value (FUV)

We used the family use value index to analyze the association between botanical families and users of taxa that correspond to these families. This index is equal to the mean total use value of each species in the family (Hoffman and Gallaher, 2007).

$$\text{FUV}=\frac{\sum UV}{N}$$

Where;

FUV is the family use value, UV is the utility value of the family's species, and N is the number 173 of species in the family.

• Informant Consensus Factor (ICF).

The ICF demonstrates the uniformity of traditional knowledge exchange amongst informants regarding the usage of plants to cure different types of diseases. The following formula was used to determine ICF (Bencheikh et al., 2021e).

$$ICF=\frac{\text{Nur}-\text{Nt}}{\text{Nur}-1}$$

Where;

Nur denotes the number of use-reports for an ailment category and Nt denotes the total number of plants used by all informants for that illnesses category. The ICF values range between 0 and 1, with values close to 0 indicating that the herbs were picked at random or that there was no exchange of information about plant usage within the population. Furthermore, ICF values close to 1 indicate a clear selection of medical species and information sharing about their use in the population.

• Fidelity Level (FL).

The level of fidelity (FL) identifies a plant species’ ability to effectively combat a certain disease. FL was determined using the formula below (Sreekeesoon and Mahomoodally, 2014).

$$FL=\frac{\text{Ip}}{\text{Lu}}*100$$

Where;

**Lu** denotes the total number of interviewers who cited all uses of the particular species for the therapies of all liver pathologies, and **Ip** represents the number of individuals who used a particular species for a specific type of liver disease.

### 2.5 Pharmacological validation

A bibliographic search was conducted to identify the biological activities of identified plants against liver disease, by the mean of the following databases: PubMed, Science Direct, Google Scholar, Scopus and Web of Science with keywords like “liver disease,” “liver disease,” “Liver failure,” “hepatitis,” “Jaundice,” and “Hepatoprotective” combined with the scientific name of each plant.

## 3 Results and discussions

### 3.1 Informants’ sociodemographic profile

A total of 197 informants, including 189 non-specialists and 8 herbalists interviewed for this study. These interviewees are spread over twelve rural stations in five provinces of North-East Morocco (Table 1). The socio-demographic profile of the participants in this study (The variable comprising age, sex, education level, income and attitude towards drugs) were grouped in the Table 2. Analysis of the data presented in Table 2 shows that Women had the highest share of participants (59%), followed by men (40.6%). The use of medicinal plants for the treatment of liver disease in the study areas is widespread in all age groups. The 46–65 age group is the most represented in this study with a frequency of 58.88%, followed by the 25–45 age group with a percentage of 25.38%, the over-65 age group with 14.21%, and the under-25 age group with a percentage of 1.52%. The results of numerous research have consistently shown that older people had more traditional knowledge on how to use medicinal herbs than did younger people (Alami Merrouni et al., 2021; Bencheikh et al., 2021e; Hachlafi et al., 2022). The discomfort of the younger generation, which tends not to accept popular medicine due to the effect of exotic culture, and the influence of lifestyle modernization can be used to explain the gradual loss of traditional knowledge about medicinal plants (Sargin et al., 2015). The fact that there were fewer informants over the age of 65 (14.21%) is a reflection of the depth of traditional knowledge being lost as rural elders pass away.

**TABLE 1:** Number of informants for each locality.

Provinces	Stations	Number of informants	
		Local residents	Herbalist
Guercif	Ras Laksar	10	0
	Saka	11	1
	Jal	16	0
Jerada	Ain Benimathar	18	0
	Guenfouda	10	2
Berkane	Tafoughalt	11	2
	Ahfir	25	0
Nador	Tiztoutine	10	0
	Bouarg	12	1
Oujda-Angad	Bni Drar	31	2
	Naima	19	0
	Sidi Moussa Lemhaya	16	0
Total	12 stations	189	8

**TABLE 2:** Socio-demographic characteristics of informants.

Distribution	Categories	Informants number	Percentage of informants
			%
By sex (197)	Men	80	40.6
	Women	117	59.4
By age range	Less than 25 years	3	1.52
	25–45	50	25.38
	46–65	116	58.88
	More than 65 years	28	14.21
By education level	Illitirate	75	38.07
	Primary education	69	35.03
	Secondary education	38	19.29
	University education	15	7.61
By income/month	Unemployed	81	41.11
	500–2000 DH	89	45.17
	2000–6000 DH	12	6.1
	>6000 DH	15	7.61
By choice of medicine	Herbal medicine	101	51.27
	Both conventional and herbal medicine	76	38.58
	Modern medicine	20	10.15

In terms of educational attainment, the findings revealed that 38.07% of the informants are illiterate, followed by the categories of secondary and primary education, with percentages, respectively 35.03% and 19.29%, and lastly the university level, with a percentage of 7.61%. These findings are consistent with those of other ethnobotanical studies conducted in various regions of Morocco (Khouchlaa et al., 2017b; Bencheikh et al., 2021e; Hachlafi et al., 2022). The study area’s rising illiteracy rate may be caused by the fact that poverty is still pervasive in the rural areas examined. This is indicated in our results, where the majority of respondents had a low socio-economic level (41.11% unemployed, and 45.17% between 500 and 2000 DH/month).

There are differences in how the people in this area feel about treating liver illness. The results shown in Table 2 demonstrate the extreme variety of usage patterns. In fact, the majority of interviews indicated that traditional medicine was their first choice of treatment when they were ill, with a percentage of 51.27%, followed by the use of conventional and herbal medicine in second place, with a percentage of 38.58%, and exclusively modern medicine in third place, with a percentage of 10.15%. Access to modern medication is hampered by a lack of health facilities and trained medical personnel, a lack of infrastructure, particularly paved roads, a lack of transportation options, a lack of logistical support, and the high expense of treating liver disease with modern medicine (El Hassani et al., 2013; Eddouks et al., 2017). The aforementioned factors all strongly encourage rural populations to switch to traditional healthcare, especially the usage of medicinal herbs.

### 3.2 Diversity of plant species used to treat liver diseases

This study recorded the use of 45 medicinal plants, spread across 26 families and 43 genera, for the treatment of liver disease in the study area. Traditional information on the applications of these plants has been developed (Table 3), including the use value, scientific name, botanical family, popular names, traditional uses, parts utilized, preparation procedure, and mode of administration for each medicinal species.

**TABLE 3:** Medicinal plants used in the study area for the treatment of liver ailments.

Botanical family Scientific name (voucher number)	Local name	Therapeutic uses	Part used	Mode of preparation	Mode of administration	UV
ASTERACEAE *Artemisia absinthum* L. (HUMPOM903)	Chiba - lahyat cheikh	Hepatic colic, hepatitis, detoxification, jaundice	Leaves	Decoction	Oral	0.0355
*Artemisia herba-alba* Asso. (HUMPOM923)	Izri - halfa	Detoxification	Leaves	Decoction	Oral	0.0051
*Cynara scolymus* L. (HUMPOM924)	Khorchef	Liver diseases, hepatic colic	Stems, flowers	Infusion	Oral	0.0203
*Anacyclus pyrethrum* (L.) Lag. (HUMPOM925)	Oud alatass	Liver diseases	Stems	Decoction	Oral	0.0051
*Reichardia intermedia* (Sch.Bip.) Samp. (HUMPOM926)	Nokad	Hepatic colic, liver congestion	Leaves	Decoction	Oral	0.0152
ALLIACEAE *Allium sativum* L. (HUMPOM927)	Thouma	Liver cancer, hepatoprotective effect, liver diseases, hepatitis	Bulb, leaves	Decoction, infusion, in food, maceration	Oral	**0.1015**
ANACARDIACEAE *Pistacia lentiscus* L. (HUMPOM895)	Dro - btam	Liver cancer, liver diseases, hepatic colic	Fruit, leaves	Decoction	Oral	0.0305
APIACEAE *Cuminum cyminum* L. (HUMPOM909)	Kamoun	Detoxification, liver diseases, jaundice, hepatitis	Seed	Decoction, infusion, powder	Oral	**0.1065**
*Apium nodiflorum* (L.) Lag. (HUMPOM928)	Zyata	Liver diseases, hepatic colic, bile problems	Leaves	Decoction	Oral	0.0254
*Pimpinella anisum* L. (HUMPOM902)	Habat hlawa - yanssoun	Hepatitis, hepatitis	Leaves, fruit	Decoction, powder, infusion	Oral	0.0152
*Coriandrum sativum* L. (HUMPOM910)	Kossber	Liver diseases	Leaves	Decoction	Oral	0.0051
APOCYNACEAE *Nerium oleander* L. (HUMPOM901)	Alili, defla	Liver diseases, liver cancer	Leaves	Decoction	Oral	0.0102
ARECACEAE *Cocos nucifera* L. (HUMPOM911)	Noix de coco	Jaundice	Fruit	-	Oral	0.0051
ASPARAGACEAE *Asparagus officinalis* L. (HUMPOM898)	Sekoum	Bile problems, liver stones, hepatitis, jaundice	Stems, leaves	Decoction, infusion, in food	Oral	**0.0558**
COMBRETACEAE *Terminalia arjuna* (Roxb. ex DC.) Wight and Arn. (HUMPOM915)	Aarjouna	Hepatic colic	Leaves	Decoction	Oral	0.0051
FABACEAE *Lupinus albus* L. (HUMPOM929)	Termass	Hepatitis, liver diseases	Fruit, leaves	Decoction, infusion	Oral	0.0254
*Ceratonia siliqua* L. (HUMPOM931)	Kharoub	Liver diseases, jaundice	Fruit	Maceration, infusion	Oral	0.0102
*Glycyrrhiza glabra* L. (HUMPOM930)	Arq souss	Liver diseases	Stems	Infusion	Oral	0.0051
PLANTAGINACEAE *Globularia alypum* L. (HUMPOM894)	Tasselgha	Liver diseases, hepatitis	Fruit, leaves, whole plant	Decoction	Oral	0.0203
IRIDACEAE *Crocus sativus* L. (HUMPOM912)	Zaafran lhor	Liver diseases	Flowers	Decoction	Oral	0.0051
LAMIACEAE *Salvia officinalis* L. (HUMPOM904)	Salmiya	Jaundice, liver diseases, hepatitis, liver cancer, detoxification	Leaves, stems	Decoction, infusion	Oral	**0.0761**
*Thymus vulgaris* L. (HUMPOM932)	Zaatar	Hepatoprotective effect, liver diseases, hepatic colic	Leaves	Decoction	Oral	0.0355
*Ocimum basilicum* L. (HUMPOM916)	Rihane - hbek	Liver diseases, jaundice, hepatitis	Leaves	Decoction, infusion	Oral	0.0355
*Rosmarinus officinalis* L. (HUMPOM919)	Azir - yazir	Hepatitis, detoxification, liver diseases	Leaves	Decoction	Oral	0.0254
*Lavandula dentata* L. (HUMPOM920)	Khzama	Liver diseases, hepatitis	Flowers, leaves	Decoction, infusion	Oral	0.0203
*Mentha pulegium* L. (HUMPOM921)	Fliyo	Hepatitis, jaundice	Leaves	Decoction	Oral	0.0152
*Mentha spicata* L. (HUMPOM913)	Naanaa	Liver diseases	Leaves	Infusion	Oral	0.0051
LAURACEAE *Laurus nobilis* L. (HUMPOM922)	Wrak sidna moussa	Hepatitis	Leaves	Infusion	Oral	0.0051
MALVACEAE *Malva parviflora* L. (HUMPOM905)	Khoubiza	Liver diseases	Leaves	Decoction	Oral	0.0051
MYRTACEAE *Syzygium aromaticum* (L.) Merr. and L.M.Perry (HUMPOM896)	Kronfol	Detoxification, liver diseases	Leaves	Decoction, infusion	Oral	0.0254
*Eucalyptus globulus* Labill. (HUMPOM933)	Eucalyptus	Liver diseases	Leaves	Oil	Oral	0.0051
OLEACEAE *Olea europaea* L. (HUMPOM908)	Zitoune	Liver diseases	Leaves, fruit	Oil, decoction	Oral	0.0152
PIPERACEAE *Piper nigrum* L. (HUMPOM914)	Flfla kehla	Hepatic colic, liver diseases, liver cancer	Fruit	Decoction, poudre infusion	Oral	0.0355
PLUMBAGINACEAE *Armeria alliacea* (Cav.) Hoffmanns. and Link (HUMPOM899)	Arq wedmi	Liver diseases, jaundice	Fruit, stems	Infusion, decoction	Oral	0.0152
POACEAE *Zea mays* L. (HUMPOM934)	Dorra, kbal	Liver diseases	Cones, fruit	Decoction, infusion	Oral	0.0102
*Hordeum vulgare* L. (HUMPOM906)	Chaair	Liver diseases	Leaves	Decoction	Oral	0.0051
POLYGONACEAE *Rumex vesicarius* L. (HUMPOM935)	Zriaat lhemida	Detoxification, liver diseases	Seeds, leaves	Infusion, decoction	Oral	0.0203
RANUNCULACEAE *Nigella sativa* L. (HUMPOM917)	Haba kahla	Liver cancer, liver diseases	Seeds	Decoction	Oral	0.0102
RHAMNACEAE *Ziziphus lotus* (L.) Lam. (HUMPOM918)	Nbeg	Jaundice, liver diseases, hepatic colic, hepatitis	Fruit, leaves	In food, maceration, infusion, decoction	Oral	**0.0457**
ROSACEAE *Agrimonia repens* L. (HUMPOM887)	Makerman	Jaundice, liver diseases	Leaves	Decoction	Oral	0.0102
*Crataegus monogyna* Jacq. (HUMPOM936)	Zaarour	Liver diseases	Leaves	Infusion	Oral	0.0051
RUTACEAE *Citrus × aurantium* L. (HUMPOM937)	Ranj	Liver diseases, liver congestion	Fruit	Decoction, maceration	Oral	0.0254
THEACEAE *Camellia sinensis* (L.) Kuntze (HUMPOM907)	Atay	Liver diseases	Whole plant	Infusion	Oral	0.0051
ZINGIBERACEAE *Zingiber officinale* Roscoe (HUMPOM938)	Skinjbir	Liver diseases, hepatic colic, jaundice	Whole plant, rhizomes	Decoction, infusion, powder	Oral	0.0254
*Curcuma longa* L. (HUMPOM939)	Kharkoum	Liver diseases, hepatitis	Whole plant	Powder	Oral	0.0102

Bold indicates the best values.

#### 3.2.1 Frequency of families and their use value

As indicated in Table 4, a total of 26 botanical families were used in rural areas of North-East Morocco for the treatment of liver pathologies. However, the families most used are Lamiaceae (7 species; 6 genera) in the first position, followed by Asteraceae (5 species; 4 genera), Apiaceae (4 species; 4 genera), Fabaceae (3 species; 3 genera), Myrtaceae, Poaceae, Rosaceae and Zingiberaceae with (3 species; 3 genera) for each. There are only one species and one genus for the other families. Similarly, the Lamiaceae, Asteraceae, and Apiaceae botanical families are the ones that are most prevalent in Mediterranean countries (Benítez et al., 2010; Savo et al., 2011). The predominance of the Asteraceae family in the traditional treatment of liver disease has already been confirmed by an ethnobotanical study carried out in the Maritime region of Togo (Kpodar et al., 2016).

**TABLE 4:** Distribution of botanical medicinal families according to species and genera. FUV: Family Use Value.

Family	Number of species	Number of genera	FUV	Family	Number of species	Number of genera	FUV
Lamiaceae	7	6	0.0305	Combretaceae	1	1	0.0051
Asteraceae	5	4	0.0162	Plantaginaceae	1	1	0.0203
Apiaceae	4	4	**0.1066**	Iridaceae	1	1	0.0051
Fabaceae	3	3	0.0135	Lauraceae	1	1	0.0051
Myrtaceae	2	2	0.0152	Malvaceae	1	1	0.0051
Poaceae	2	2	0.0076	Oleaceae	1	1	0.0152
Rosaceae	2	2	0.0076	Piperaceae	1	1	**0.0355**
Zingiberaceae	2	2	0.0178	Plumbaginaceae	1	1	0.0152
Alliaceae	1	1	**0.1015**	Polygonaceae	1	1	0.0203
Anacardiaceae	1	1	**0.0305**	Ranunculaceae	1	1	0.0102
Apocynaceae	1	1	0.0102	Rhamnaceae	1	1	0.0457
Arecaceae	1	1	0.0051	Rutaceae	1	1	0.0254
Asparagaceae	1	1	**0.0558**	Theaceae	1	1	0.0051

Bold indicates the best values.

Families with high FUV are Apiaceae (0.1066), Alliaceae (0.1015), Asparagaceae (0.0558), and Piperaceae (0.0355) (Table 4). However, there aren't many species in these groups to represent them. It appears that the value of using ethnobotanical families is not dependent on their particular wealth but rather on the significance and value of the use of the individual species (Najem et al., 2019). Additionally, these families’ significant FUV would be mostly dependent on their abundance of bioactive compounds, which would confer multiple benefits such as antimicrobial, anti-allergic, anti-oxidant, and anti-inflammatory properties (Bencheikh et al., 2022).

#### 3.2.2 Most used plants species to treat liver diseases according to use value index

In this work, we inventoried 45 different medicinal plants that are utilized to treat liver ailments in rural areas of North Eastern Morocco. Nevertheless, the most widely used plants for the treatment of liver diseases are *Cuminum cyminum* L. (UV = 0.1065), followed by *Allium sativum* L. (UV = 0.1015), *Salvia officinalis* L. (UV = 0.0761), *Asparagus officinalis* L. (UV = 0.0558), and *Ziziphus lotus* (L.) Lam. (UV = 0.0457) (Table 3). These five species made up 27.84% of all use ratios, while the other 40 species only made up 72.16% of all use ratios. Similar studies conducted in other nations have shown that high utilization values have been attained for plants other than those in the current study (Kotoky and Das, 2008; Kpodar et al., 2016). This difference in species similarity could be explained by the difference in bioclimate between countries, which will favor the difference in the abundance of certain plant species from one country to another. In addition, geographic distance between countries has a direct impact on the traditional cultures of indigenous peoples, as evidenced by Alami Merrouni et al. (2021), in which they demonstrated that the increase in distance between countries is accompanied by the increase in the difference in the cultures of these countries and *vice versa*. Thus, all these factors can lead to differences between countries in the use of plant species to treat a particular health condition.

These five medicinal plants were frequently utilized in traditional Moroccan medicine to cure a wide range of illnesses:

*Cuminum cyminum* L.: This Apiaceae family medicinal plant was one of the first plants grown in Asia, Africa, and Europe (Al-snafi, 2017). Since antiquity, *C. cyminum* seeds have persisted in popularity as culinary seasonings and are widely utilized in folk therapy across a variety of geographic regions. This plant, called in Morocco as “Kammun”, is frequently used conventionally to treat digestive system issues, including diarrhea (Jamila and Mostafa, 2014). According to the analysis of the data collected during our investigation, *C. cyminum* is the most widely used to treat liver pathologies in the North-Eastern Moroccan population with a usage value of 0.1065. Indeed, the seeds of *C. cyminum*, in decoction or infusion, are used by the study population as treatment of jaundice, and hepatitis, and thus for liver detoxification. In Ayurveda (former Indian therapeutic system), seeds of *C. cyminum* are traditionally used against jaundice and to improve liver function (Andallu and Ramya, 2007; Johri, 2011).

*Allium sativum* L.: This plant, called locally as “Thouma” in Morocco, is one of the earliest known to have been cultivated (Thomson and Ali, 2003). Traditional Moroccan medicine makes extensive use of garlic to cure and prevent a wide range of illnesses, including cancer, lung disease, hypertension, diabetes, microbiological infections, infertility, and problems with the kidneys (Fakchich and Elachouri, 2021). According to the results of our investigation, this plant is classified according to its use value in the second position as the plant most used to treat liver diseases in the northeast of Morocco. Indeed, the leaves and bulb of this plant in decoction or infusion are widely recommended by the inhabitants of the study area to fight against liver cancer and hepatitis, and thus declared that it has hepatoprotective effects. Furthermore, it has been reported that portions of this plant are commonly used to heal liver problems in Togo’s Maritime region (Kpodar et al., 2016). The bulb of plant is often used to treat jaundice in the southern region of Algeria (Bendaif et al., 2021).

*Salvia officinalis* L.: This round perennial shrub belongs to the Lamiaceae family and is called to as “Salmiya” in the Oriental area of Morocco. It is indigenous to the Middle East and the Mediterranean, although it has since become naturalized everywhere (Ghorbani and Esmaeilizadeh, 2017). In Morocco, the aerial part of *S. officinalis* is used to handle gastrointestinal problems, metabolic disorders, and renal ailment (Bencheikh et al., 2021e; Fakchich and Elachouri, 2021). Based on the findings of the current investigation, this plant is classified among the three most used medicinal species in the study area for the treatment of liver diseases. Indeed, the leaves and stems of *S. officinalis*, in decoction or infusion are widely used in rural areas of north-eastern Morocco to prevent and treat jaundice, hepatitis, and liver cancer, and thus to detoxify the liver. In the middle Oum Rbia region of Morocco, leaves and whole plant decocted were used for liver problems (Ben Akka et al., 2019). In addition to these local uses, in traditional South-West Algerian medicine, the flowers of this plant were also used to treat liver symptoms (Benarba, 2016).

*Asparagus officinalis* L.: Since ancient times, asparagus, a perennial herbaceous plant of the Asparagaceae family, has been utilized extensively in food and medicinal. This plant is called « Sekoum» in Morocco, is used to treat various ailments such as respiratory diseases, digestive problems, kidney diseases, liver diseases and diabetes (Alami Merrouni and Elachouri, 2021; Fakchich and Elachouri, 2021; Bencheikh et al., 2022). In our study, asparagus is ranked fourth among the most cited plants for the treatment of liver patients. In fact, this plant’s leaves and stems are frequently used to treat biliary issues, liver stones, hepatitis, and jaundice.

*Ziziphus lotus* (L.) Lam.: The majority of Africa, numerous Asian nations, including China, Iran, and South Korea, as well as several European nations, including Cyprus, Spain, and Greece, are all home to this medicinal plant (Adeli and Samavati, 2014; Bencheikh et al., 2021d). In Morocco, *Z. lotus* is locally known as “*Sedra*,” and “*Nbeg*” for its fruits, and is widely found in arid and semi-arid areas (Bencheikh et al., 2019). Plant parts were traditionally used to combat various health problems such as sedation, anxiety, urinary problems, diabetes, skin infections, scarring, and bronchitis (Khouchlaa et al., 2017a; Bencheikh et al., 2021e; Fakchich and Elachouri, 2021). As per the findings of our survey, *Z. lotus* in rural parts of North-East Morocco, is one of the top five plants used to treat liver disorders such as jaundice, hepatic colic, and hepatitis. Furthermore, the fruits of this medicinal plant are traditionally used to treat lung diseases, jaundice, and as an emollient in El Hammadia, Algeria (Bendaif et al., 2021).

### 3.3 Ethnic medicinal characteristics

**FIGURE 2:**
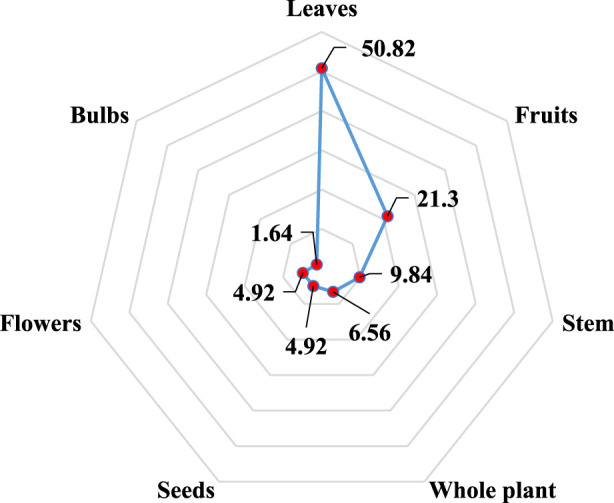
Distribution of plant parts by the percentage of use (%).

In this study, different parts of plants are used as medicines to treat liver problems in rural areas of North-East Morocco (Figure 2). Thus, on the basis of calculating the percentage of use of each part (%), the leaves (50.82) appear to be the most commonly utilized for the treatment of liver illnesses in the study area, followed by fruits (21.3), stems (9.84), whole plants (6.56), the seeds, and the flowers (4.92) for each, and finally the bulbs (1.64). The leaves are both a source of photochemical reactions and a repository of organic stuff created from them, which explains why they are used so frequently (Bencheikh et al., 2021e). In addition, it is important to avoid pulling out the entire plant or picking up the roots of the plants, as this will promote deforestation and put the species at risk (Kadir et al., 2013). On the contrary, the use of leaves contributes to the conservation and sustainable use of the plant.

**FIGURE 3:**
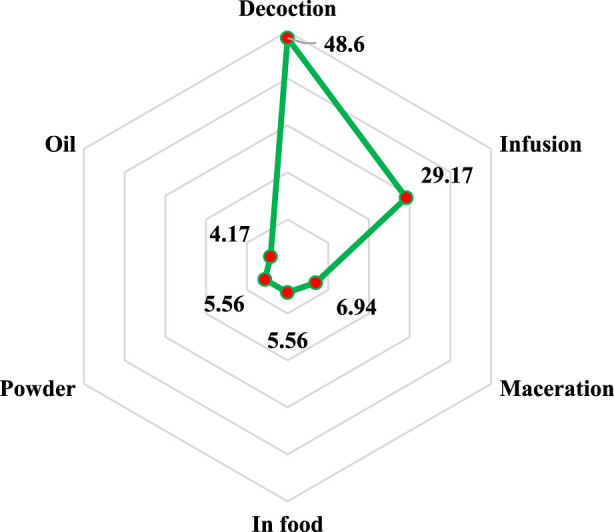
Distribution of preparation methods according to percentages of use.

As seen in Figure 3, various techniques are used in rural North-East Morocco to make alternative therapies for treating liver disease. Nevertheless, with a percentage of 48.61%, decoction remains the most commonly employed method of preparation, followed by infusion (29.17%), maceration (6.94%), powder and preparation in the diet (5.56% for each), and finally oils with 4.17%. The preparation technique is frequently correlated with the type of use (external or internal); typically, external usage involves the use of a mask, massage, or suppositories, while internal use involves the use of decoction, infusion, maceration, and other techniques (Eddouks et al., 2017). Decoction’s supremacy may thus be explained by the fact that it allows for the capture of the greatest amount of bioactive molecules and reduces or eliminates the toxic effects of some recipes (Noureddine et al., 2022).

### 3.4 Hepatic ailments categories and their informant consensus factor (ICF) values

**FIGURE 4:**
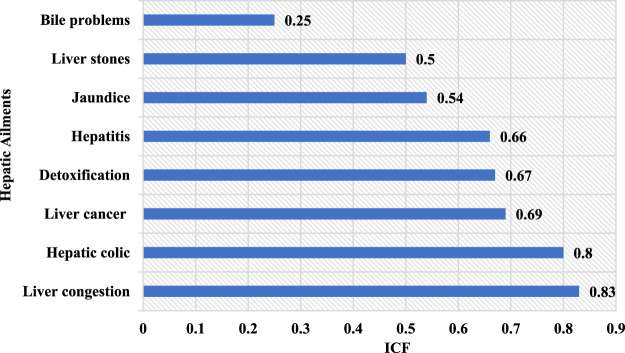
Category of hepatic illness and their informant consensus factor (ICF).

**FIGURE 5:**
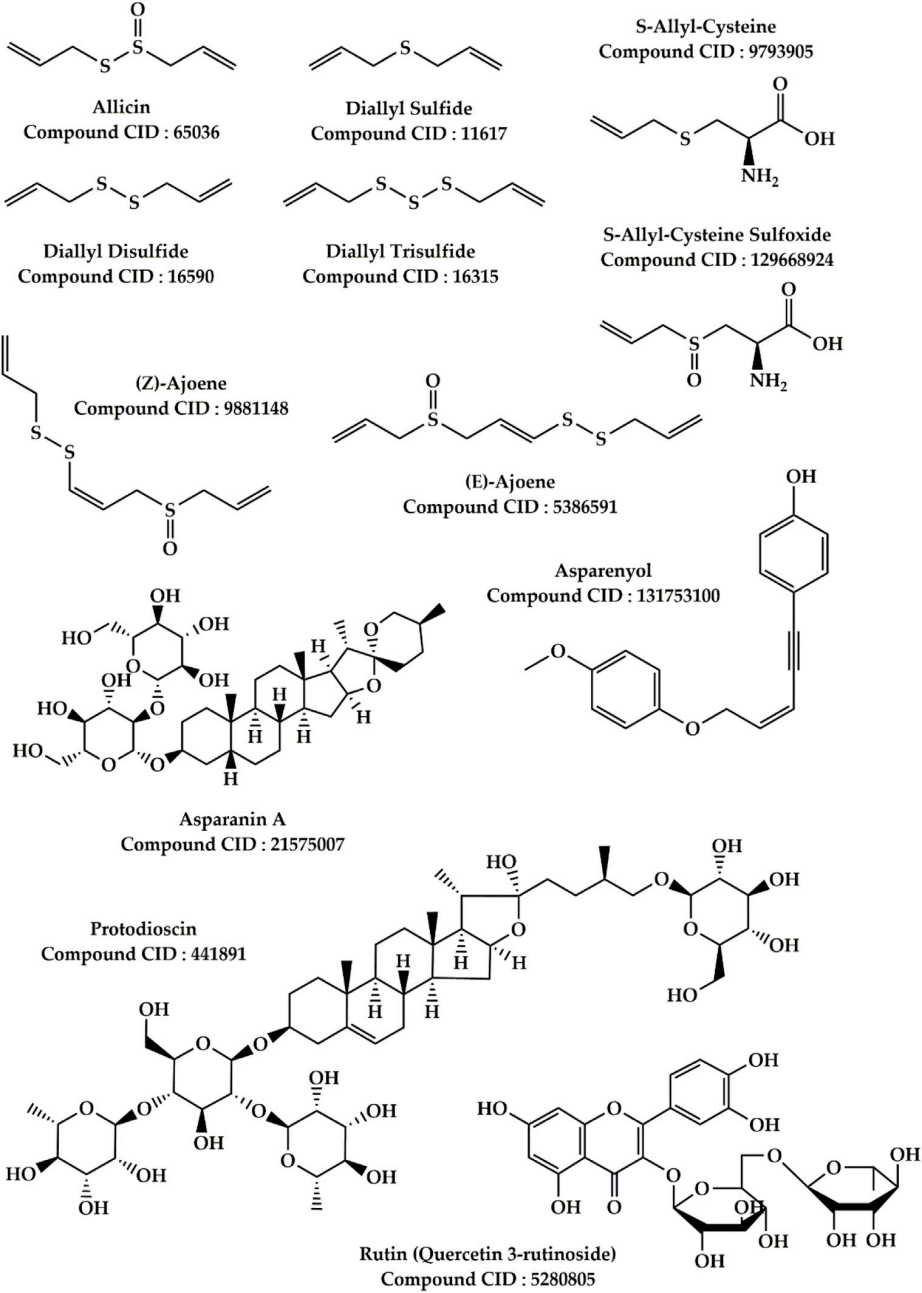
Main components found in *Allium sativum* L. and *Asparagus officinalis* L.

In this study, we identified eight liver pathologies that were treated with medicinal plants in rural areas of North-East Morocco (Figure 4). The ICF values of the plant species cataloged in this investigation extended from a minimum of 0.25 to a maximum of 0.83. (Figure 5). This index has the highest value for liver congestion (ICF = 0.83), followed by hepatic colic (ICF = 0.80), liver cancer (ICF = 0.69), liver detoxification (ICF = 0.67), hepatitis (ICF = 0.66), jaundice (ICF = 0.54), liver stone (ICF = 0.50), and bile problems with ICF = 0.25. High values (around 1) of this index for liver congestion, hepatic colic, and liver cancer suggest that a small number of species were employed by many informants, reflecting a high level of consensus on the use of plants in the management of these illnesses. The low accord between both interviews was witnessed for biliary problems. This could be attributed to a lack of interaction and knowledge exchange among individuals (Al-Qura’n, 2005).

### 3.5 Fidelity level (FL)

According to the corresponding level of fidelity, we categorized the medicinal plants used to treat liver illness in Table 5. According to our findings, the level of fidelity of plant species for a particular liver condition ranged between 9.09% and 100%. Concerning hepatitis problems, the most important species according to the level of fidelity were *Pimpinella anisum* L. (FL = 100%), *Laurus nobilis* L. (FL = 100%), *Lavandula dentata* L. (FL = 75%), and *Lupinus albus* L. (FL = 66.67%). For the jaundice, *Cocos nucifera* L. (FL = 100%), *Mentha pulegium* L. (FL = 100%), *Z. lotus* (L.) Lam. (FL = 66.67%), and *Agrimonia repens* L. (FL = 66.67%) were the most important. The most widely known species in the hepatic colic group were *Terminalia arjuna* (Roxb. ex DC.) Wight & Arn. (FL = 100%), and *Piper nigrum* L. (FL = 60%). For liver detoxification, plants with the highest FL were *Syzygium aromaticum* (L.) Merr. & L.M. Perry (FL = 33.33%), *Rumex vesicarius* L. (FL = 33.33%), and *Artemisia herba-alba* Asso (FL = 28.57%). *Nerium oleander* L. (FL = 66.67%), and *A. sativum* L. (FL = 50%) were species with the highest fidelity level. For liver congestion, *Citrus × aurantium* L. (Fl = 66.67%) was the most important. In the end, *Asparagus officinalis* L. is the most important for liver stone and bile problems. The importance of these plants for the treatment of liver diseases in the study area could be due to their wide use in traditional Moroccan medicine to treat various diseases (Fakchich and Elachouri, 2021).

**TABLE 5:** Fidelity level values of medicinal plants for each category of liver illness.

Category of illness	Name of species	Fidelity level (FL %)
Hepatitis	*Pimpinella anisum* L	**100.0**
	*Laurus nobilis* L	**100.0**
	*Lavandula dentata* L	75.00
	*Lupinus albus* L	66.67
	*Artemisia absinthium* L	50.00
	*Rosmarinus officinalis* L	50.00
	*Globularia alypum* L	40.00
	*Mentha pulegium* L	33.33
	*Curcuma longa* L	33.33
	*Cuminum cyminum* L	25.00
	*Salvia officinalis* L	25.00
	*Ocimum basilicum* L	25.00
	*Asparagus officinalis* L	16.67
	*Allium sativum* L	12.50
Jaundice	*Cocos nucifera* L	**100.0**
	*Mentha pulegium* L	**100.0**
	*Ziziphus lotus* (L.) Lam	66.67
	*Agrimonia repens* L	66.67
	*Ocimum basilicum* L	50.00
	*Cuminum cyminum* L	37.50
	*Armeria alliacea* (Cav.) Hoffmanns. and Link	33.33
	*Salvia officinalis* L	25.00
	*Zingiber officinale* Roscoe	25.00
	*Ceratonia siliqua* L	20
	*Asparagus officinalis* L	16.67
	*Artemisia absinthium* L	13.64
Hepatic colic	*Terminalia arjuna* (Roxb. ex DC.) Wight and Arn	**100.0**
	*Piper nigrum* L	60.00
	*Reichardia intermedia* (Sch.Bip.) Samp	50.00
	*Thymus vulgaris* L	50.00
	*Zingiber officinale* Roscoe	50.00
	*Cynara scolymus* L	37.50
	*Pistacia lentiscus* L	37.50
	*Apium nodiflorum* (L.) Lag	37.50
	*Artemisia absinthium* L	27.27
Detoxification	*Syzygium aromaticum* (L.) Merr. and L.M.Perry	**33.33**
	*Rumex vesicarius* L	**33.33**
	*Artemisia herba-alba Asso*	28.57
	*Cuminum cyminum* L	25.00
	*Rosmarinus officinalis* L	25.00
	*Artemisia absinthium* L	9.09
Liver cancer	*Nerium oleander* L	**66.67**
	*Allium sativum* L	50.00
	*Pistacia lentiscus* L	37.50
	*Nigella sativa* L	33.33
	*Salvia officinalis* L	25.00
	*Piper nigrum* L	20.00
Liver congestion	*Citrus × aurantium* L	**66.67**
	*Reichardia intermedia* (Sch.Bip.) Samp	50.00
Liver stones	*Asparagus officinalis* L	**16.67**
Bile problems	*Asparagus officinalis* L	**50.00**

Bold indicates the best values.

### 3.6 Pharmacological confirmation data of the medicinal plants

The current ethnobotanical fieldwork confirmed that the inhabitants of northeastern Morocco has extensive ethnobotanical information concerning the use of herbal remedies in the treatment of liver conditions. These conventional data, which detailed a wide variety of quantitative factors, were particularly intriguing for the goal of bioprospecting to identify novel drugs to cure liver pathological conditions. It could be worthwhile to look up these plants’ pharmacological properties in the literature. According to the results of our bibliographic survey, of the 46 plant species registered for the treatment of liver diseases in the study area, 28 plant species from 20 botanical families have already been pharmacologically validated for liver diseases (Table 6). It can be concluded that the majority of them significantly reduce the risk of liver disorders. These findings demonstrated the potential of ethnobotanical knowledge as a preferable traditional database for plant species with beneficial therapeutic effects connected to liver illnesses. The pharmacological data collected for the plants selected during our survey were grouped in the Table 6.

**TABLE 6:** Pharmacological outcomes of medicinal plants cited by locals to cure liver ailments.

Scientifique name	Used parts	Used extracts	Experimental model	Pharmacological uses	Therapeutic doses	Ref.
*Allium sativum* L	Leaves or bulbs	Aqueous extract	Rats	Hepatoprotective effect against acute ethanol-induced oxidative stress in rat liver	250 mg/kg of b.w (for 5 days)	Nencini et al. (2010)
	Garlic cloves	Ethanol extract	HepG2 cells	Cytoprotection against mycotoxins on HepG2 cells	1% during 24 and 48 h	Juan-García et al. (2021a)
	Bulbs	Aqueous extract	Rats	Protective effect on alloxan-induced elevations of plasma biochemical factors of hepatic functions	100 or 200 mg/kg of b.w/day (for 21 days)	Aprioku and Amah-Tariah (2017a)
*Artemisia absinthium* L	Aerial parts	Aqueous extract	Mice	Protective effect against CCl_4_ and endotoxin-caused liver damage	50–200 mg/kg of b.w (for 7 days)	Amat et al. (2010)
	Aerial parts	Hydroalcoholic extract	Rats	Reduce serum levels of ALT, AST, and oxidative damage in rats to alleviate liver toxicity	100 mg/kg of b.w (for 24 h)	Mohammadian et al. (2016)
	Aerial parts	Ethanol extract and its fractions, petroleum ether, and ethyl acetate	Human hepatoma BEL-7404 cells, and mouse hepatoma H22 cells	Induce apoptosis in Hepatocellular carcinoma cells via the endoplasmic reticulum stress and mitochondrial-dependent pathways to inhibit cell growth	25–150 μg/mL (for 24, 48, and 72 h)	Wei et al. (2019)
	Leaves	Methanol and ethyl acetate extract	Rats	Hepatoprotective effect against diclofenac-induced liver toxicity in rats	50–200 mg/kg of b.w/day (for 5 days)	Antonio et al. (2020)
*Asparagus officinalis* L	Asparagus spears	Ethanolic and aqueous extracts	Mice	Prevent liver against high-fat diet	200 mg/kg of b.w (for 10 weeks)	Zhu et al. (2010)
	Roots	Hydroalcoholic extract	Rats	Prevent the liver from oxidative stress enhanced by cadmium chloride	100–400 mg/kg of b.w (for 28 days)	Abedi et al. (2018)
*Ceratonia siliqua* L	Leaves	Aqueous extract	Mouse hepatocellular carcinoma cell line (T1)	Anti-cancer effect against hepatocellular carcinoma cell line	0.2–0.4 mg/mL (for 24 h)	Corsi et al. (2002)
	Leaves	Hydroethanolic and ethyl acetate extracts	Rats	Protective effect against hepatotoxicity caused by CCl_4_ in rats	250 mg/kg of b.w (for 8 days)	Hsouna et al. (2011)
	Pods	Aqueous extract	Mice	Improves liver fibrosis caused by *Schistosoma mansoni*	300–600 mg/kg of b.w (daily for 10 days)	Al-Olayan et al. (2016)
	Pods	Aqueous extract	Rats	Hepatoprotective effect against dextran sulfate sodium in rats	50–100 mg/kg of b.w (for 14 days)	Rtibi et al. (2016)
	Seeds	Powder	Rats	Protective effects on ethanol-iduiced hepatotoxicity in rats	15% in diet supplementation	Temiz et al. (2015)
*Cocos nucifera* L	Inflorescences	Acetone extract	Rats	Protective effect on acetaminophen-caused hepatotoxicity in rats	100–400 mg/kg of b.w (for 14 days)	Chithra et al. (2020)
	Unspecified	Essential oils	Rats	Improved hypothyroidism by the reduction of liver functions	10% in diet (for 6 weeks)	Mohammed et al. (2020)
*Coriandrum sativum* L	Fruits	Essential oils	Mice	Hepatoprotective effect against CCl_4_-induced liver damage	0.13 g/kg of b.w (during 5 consecutive days)	Samojlik et al. (2010)
	Fruits	Essential oils	Rats	Hepatoprotective effect against liver toxicity-induced by ibuprofen in rats	40 mg/kg of b.w (for fourteen consecutive days)	Baghdadi et al. (2016)
	Leaves and seeds	Coriander sauces	Rabbits	Hepatoprotective effect against CCl_4_-induced toxicity in rabbits	15 mL/kg of b.w	Iqbal et al. (2018)
	Areal parts and seeds	Aqueous extract	Rats	Hepatoprotective effect against hepatic injury provoked by lambda-cyhalothrin insecticide	1% (w/w) in died (for 90 days)	Boutlelis et al. (2020)
*Crataegus monogyna* Jacq	Fruits	Aqueous extract	Rats	Exhibits a protective effect on doxorubicin-induced the liver toxicity	20 mg/kg of b.w (for 28 days)	Shalizar-jalali et al. (2013)
*Crocus sativus* L	Stigmas	Aqueous extract	Rats	Protective effects against chronic-stress induced oxidative damage liver in rats	30 mg/kg of b.w (daily for 21 days)	Bandegi et al. (2014)
	Flowers	Aqueous extract	Rats	Alleviate methotrexate-induced liver toxicity in rats	80 mg/kg of b.w (for 10 days)	Hoshyar et al. (2019)
	Stigmas	Ethanolic extract	Rats	Protective effect on oxidative damages in aged male rat liver	5–20 mg/kg of b.w (daily for 4-week)	Samarghandian et al. (2016)
	Petals and stigmas	Aqueous and ethanolic extracts	Rabbits	Hepatoprotective effect on amiodarone-provoked liver toxicity in rabbits	100 mg/kg of b.w (for 3 days)	Riaz et al. (2016)
	Stigmas	Ethanolic extract	Rats	Beneficial effect for the liver	0.35 g/kg of b.w (daily for 2 weeks)	Mohajeri et al. (2007)
	Petal	Hydroalcoholic extract	Rats	Anti-inflammatory effect in liver toxicity caused by alcohol consumption	167.5–335 mg/kg of b.w (for 8 weeks)	Azizi et al. (2019)
	Petals	Hydroalcoholic extract	Rats	Hepatoprotective effect against acetaminophen provoked liver toxicity in male rats	10–20 mg/kg of b.w (daily for 6 days)	Omidi et al. (2014)
	Tepals, stigmas and leaves	Hydroethanolic extract	Rats	Protective effects on CCl_4_-provoked acute liver toxicity in rats	50 mg/kg of b.w (daily for 14 days)	Ouahhoud et al. (2021a)
*Curcuma longa* L	Rhizomes	Ethanolic extract	Rats	Prevent on thioacetamide-provoked liver cirrhosis in rats	250–500 mg/kg of b.w (for 8 weeks)	Salama et al. (2013)
	Rhizomes	Ethanolic extract	Chicken	Hepatoprotective effect against aflatoxine-causing liver toxicity	5 mg/kg of b.w (for 28 days)	Gholami-ahangaran et al. (2016)
	Rhizomes	n-Hexane extract	Rats	Protective effect on hepatotoxicity induced by ethanol	200 mg/kg of b.w (for 28 days)	Nwozo et al. (2014)
	Rhizomes	Aqueous extract	Mice	Inhibiting hepatic oxidative stress and inflammatory cytokine secretion in ethanol-induced liver injury	20 mg/kg of b.w (unspecified)	Uchio et al. (2017)
	Rhizomes	Hydro-alcoholic extract	Rats	Inhibiting hepatic oxidative stress in adriamycin-provoked liver toxicity	100 mg/kg of b.w (for 4 weeks)	Article et al. (2024)
	Unspecified Unspecified		Rats	Protective effect on the liver toxicity provoked by AlCl_3_	40 mg/kg of b.w (for 8 weeks)	Khouja (2017)
	Rhizomes	Ethyl acetate extract	Rats	Prevent the liver on alcohol-caused hepatotoxicity in rats	100–350 mg/kg of b.w (for 14 days)	Antiya et al. (2021)
*Cynara scolymus L*	Leaves	Aqueous extract	Rats	Inhibits cholesterol production in hepatocytes	0.1–4.0 mg/mL	Gebhardt (1998)
	Leaves	Ethanol extract	Rats	Prevent liver on CCL_4_-caused oxidative stress and liver damage	1.5 g/kg of b.w (for 2 weeks)	Colak et al. (2016)
	Buds	Methanolic extracts	Rat Hepatocytes and on Human Hepatoma Cells	*In vitro* protection against oxidative stress in hepatocytes	400–1,200 µM (for 24, 48, and 72 h)	Miccadei et al. (2008)
	Leaves	Ethanol extract	Rats	Hepatoprotective effect against obesity	200–400 mg/kg of b.w (during 2 months daily)	Salem et al. (2019)
	Receptacle and bracts	Petroleum ether and ethyl acetate extracts	Rats	Reduced liver tissue lesions when damaged by thioacetamide	1.5 g/kg of b.w (daily for 2 months)	El-Mesallamy et al. (2020)
	Roots	Hydro-alcoholic extract	Rats	Prevent liver on CCL_4_-provoked hepatotoxicity	300–900 mg/kg of b.w (for 3 days)	Huseini et al. (2011)
	Leaves	Hydroethanolic extract	Rats	Inhibit hepatic oxidative stress in hepatotoxicity induced by diazinon	1,500 mg/kg of b.w (for 15 days)	Ahmadi et al. (2019)
	Leaves	Aliphatic alcohols extract	HepG2 liver cells	Increased the mitochondrial dehydrogenase activities of the human liver HepG2	100 mg/mL for 48 h	Löhr et al. (2009)
	Leaves	Aqueous extract	HepG2 cells	Against genotoxicity of HepG2 cells, and modulate hydrogen peroxide DNA damage	0.62–5.0 mg/mL (for 1 h)	Pereira et al. (2017)
	Leaves	Unspecified	Mice	Prevent the liver against obesity	5% in fied (for 1 month)	Azeem et al. (2016)
*Glycyrrhiza glabra L*	Roots	Aqueous extract	Rabbits	Protective effect on CCL_4_-caused acute liver toxicity	2 g/kg of b.w (for 7 days)	Al-Razzuqi et al. (2012)
	Roots	Aqueous and ethanol extracts	Rats	Protective effect on CCl_4_-caused acute liver toxicity	250–500 mg/kg of b.w	Laylani (2016)
	Roots	Ethanolic extract	Rats	Prevent the liver against paracetamol provoked liver acute toxicity	200 mg/kg of b.w (once a day for 7 days)	Tajua et al. (2011)
	Roots	Aqueous extract	Rabbits	Protective effect on CCl_4_ induced hepatotoxicity	2 g/kg of b.w (daily for 7 days)	Omar and Omar (2014)
	Roots	Methanolic extract	HepG2	Prevent the HepG2 cell line against H_2_O_2_	10–100 μg/mL	Shinde et al. (2016)
	Unspecified	Unspecified	Fish hepatocytes (*Cyprinus carpio*)	Hepatoprotective effect against CCl_4_-induced hepatocyte damage in common carp	2.5–10 **μ**g/mL (for 4 h)	Yin et al. (2011)
	Roots	Hydromethanolic extract	Mice	Hepatoprotective effect against CCl_4_ induced oxidative-stress mediated hepatotoxicity	300 mg/kg of b.w (once a day up to 7days)	Sharma and Agrawal (2014)
*Hordeum vulgare* L	Seeds	Unspecified	Rats	Lowering hyperlipidemia and improving liver enzymes and nearly restoring tissues of the liver to their normal structure	10% in died (for 8 weeks)	Abulnaja and Rabey (2015)
	Seeds	Methanolic extract	Rats	Prevent the liver on acetaminophen caused liver toxicity	300–500 mg/kg of b.w (for 3 days)	Pa et al. (2010)
	Seeds	Methanolic extract	Rats	Hepatoprotective effect on ethanol-provoked hepatotoxicity	300–500 mg/kg of b.w (for 18 days)	Shah et al. (2009)
*Laurus nobilis* L	Leaves	Etahanol extarct	Rats	Protective effect on CCl_4_ provoked liver toxicity	0.2 mL per 100 g rat mass	Gasparyan et al. (2015)
	Leaves	Etahanol extarct	Rats	Hepatoprotective on sodium valproate-caused liver damage	150 mg/kg of b.w (for 30 days)	Mahdi et al. (2022)
	Leaves	Methanol extarct	Rats	Prevent liver against paracetamol caused hepatotoxicity	200–400 mg/kg of b.w (for 7 days)	Ravindran et al. (2013)
*Lavandula dentata* L	Leaves	Aqueous extract	Mice	Hepatoprotective effect against thioacetamide provoked hepatic fibrosis in mice	200 mg/kg of b.w (for 8 weeks)	Almalki (2022)
*Malva parviflora* L	Whole plant	Methanol extarct	Mice	Hepatoprotective effect against paracetamol- provoked hepatotoxicity in mice	250 mg/kg of b.w (for 7 days)	Mallhi et al. (2014)
*Mentha spicata* L	Aerial parts	Aqueous extract	Rats	Protective effects against nicotine-induced toxicity in liver of rat	100 mg/kg of b.w (for 2 months)	Ben Saad et al. (2018)
*Nigella sativa* L	Seeds	Essential oils	Mice	Protective effect on the liver injury provoked by *Schistosoma mansoni*	2.5 and 5 mL/kg (for 2 weeks)	Mahmoud et al. (2002)
	Seeds	Aqueous extract	Rats	Protective effect on CCl_4_-caused liver toxicity	250–500 mg/kg of b.w (for 5 days)	Al-ghamdi (2015)
	Seeds	Essential oils	Rats	Hepatoprotective effects in CCl_4_-treated rats	0.2 mL/kg (for 60 days)	Kanter et al. (2005)
	Seeds	Unspecified	Patients with non-alcoholic fatty liver disease	Improves biochemical and fatty liver changes in non-alcoholic fatty liver disease patients	1g twice a day for 3 months	Hussain et al. (2017)
	Seeds	Aqueous extract	Mice	Protective effect against N-acetyl-p-aminophenol-induced injury in male mice	0.25 g/kg of b.w (for 30 days)	Hamza and Al-harbi (2015)
	Unspecified	Essential oils	Rats	Protective effects on carboplatin-provoked hepatotoxicity	4 mL/kg	Erisgin et al. (2019)
	Unspecified	Essential oils	Rats	Prevent the liver lesions induced by irradiation	2 mg/kg for 4 weeks	Radwan and Mohamed (2018)
	Seeds	Hydroethanolic extract	Rats	Prevent liver injury on lipopolysaccharide-provoked hepatotoxicity	100–400 mg/kg of b.w (for 2 weeks)	Rats et al. (2018)
	Seeds	Essential oils	Rats	Prevent liver against ethanol provoked oxidative stress and hepatotoxicity	2.5–5.0 mL/kg of b.w (for 3 weeks)	Develi et al. (2014)
	Unspecified	Essential oils	Rats	Prevent against aluminium chloride-provoked liver damage	2 mL/kg of b.w (once per day for 5 weeks)	Bouasla et al. (2014)
	Unspecified	Essential oils	Rats	Prevent the liver lesions induced by irradiation	1 g/kg of b.w (for 10 days)	Cikman et al. (2014)
	Seeds	Aqueous extract	Rats	Protective and restorative impact on cholestatic liver failure in bile duct ligated rats, possibly via reduced neutrophil infiltration and oxidative stress in hepatocytes	0.2 mL/kg of b.w (for 14 days)	Coban et al. (2010)
	Unspecified	Essential oils	Rats	Improves cisplatin’s effect on membrane enzymes, carbohydrate metabolism, and reactive oxygen species in liver	2 mL/kg of b.w (for 14 days)	Farooqui et al. (2016a)
	Unspecified	Essential oils	Rats	Hepatoprotective Effect on CCl_4_ caused liver toxicity in adult rats	2–4 mL/kg of b.w (for 2 weeks)	Danladi et al. (2013)
	Seeds	Unspecified	Mice	Protective effect against Dimethylaminoazobenzene provoked liver carcinogenesis in mice	5% (for 32 weeks)	Mohamed et al. (2011)
	Unspecified	Essential oils	Rats	Protective effect in thioacetamide- provoked liver cirrhosis in albino rat	5 mL/kg of b.w (for 8 weeks)	Nehar and Kumari (2013)
	Seeds	Hydroalcoholic extracts	Rats	Protective effect on CCl_4_-caused hepatotoxicity	400–800 mg/kg of b.w (for 3 days)	Kingsley (2020)
	Unspecified	Essential oils	Rabbits	Protective effect on CCl4-caused hepatotoxicity	0.2 mL/kg of b.w (for 7 days)	Al-razzuqi et al. (2011)
	Seeds	Aqueous extract	Rats	Protective effect on rifampicin caused hepatotoxicity	2 g/kg of b.w (for 28 days)	Al-azzawi and Baraaj (2016)
	Seeds	Aqueous extract	Rats	Protective effect on thioacetamide-provoked liver fibrosis	50 mg/kg of b.w (for 7 weeks)	Salem et al. (2017)
	Unspecified	Essential oils	Mice	Protective effect on diclofenac sodium and ibuprofen provoked hepatotoxicity	2.5 mL/kg of b.w	Husna and Sajjad (2017)
	Seeds	Unspecified	Chicken	Hepatoprotective effect on aflatoxin-caused liver toxicity	1%	Abosaleh et al. (2019)
	Unspecified	Essential oils	Rats	Reduced the hepatotoxicity provoked by ochratoxin A	0.3 mL/kg of b.w (for 4 weeks)	Alhussaini (2015a)
	Unspecified	Essential oils	Rats	Protective effect on thioacetamide-provoked liver toxicity	10 mL/kg of b.w (for 6 days)	Tanbek et al. (2017)
	Seeds	Unspecified	Rabbits	Protective effect in isoniazid-induced liver toxicity in rabbits	500–1,000 mg/kg of b.w (for 20 days)	Panezai et al. (2022)
	Seeds	Hydro-alcoholic extract	Mice	Protective effect against diethyl phthalate induced changes in mitochondrial enzymatic activities in liver of mice	150–300 mg/kg of b.w (for 30 days)	Prajapati and Verma (2022)
	Seeds	Unspecified	Mice	Protective effect against CCl_4_ provoked liver injury in mice	4 mL/kg of b.w (for 3 weeks)	Aleem et al. (2020)
*Ocimum basilicum* L	Leaves	Aqueous and ethanol extracts	Rats	Hepatoprotective effect on CCl_4_-induced liver fibrosis in rats	200 mg/kg of b.w (for 6 weeks)	Yacout et al. (2012)
	Leaves	Petroleum ether, chloroform, alcohol and Aqueous extracts	Goat liver	Hepatoprotective effect against H_2_O_2_ and CCl_4_ induced hepatoxicity in goat liver	100 mg/kg of b.w (for 5 days)	Meera et al. (2009)
	Unspecified	Essential oils	Rats	Modulates hematotoxicity, reactive oxygen species ( ros, DNA damage, and cell cycle arrest caused by β-cyfluthrin in rat liver	3 mL/kg of b.w (every day for a month)	Jebur et al. (2022)
	Leaves	Aqueous extract	Rats	Prevent liver on methotrexate-caused hepatotoxicity	1% (for 42 days)	El Shahat et al. (2017)
	Leaves	Aqueous extract	Rats	Prevent hepatic damage caused by arsenic	400 mg/kg of b.w (once a day for 5 weeks)	Osman et al. (2020)
	Leaves	Aqueous extract	Rats	Hepatoprotective effect on adriamycin-caused liver toxicity	20 mg/kg of b.w (for 8 weeks)	Bayomy et al. (2016)
	Whole plant	Chloroform, diethylether, ethylacetate and methanol extracts	Rats	Hepatoprotective effect on acetaminophen-provoked liver injury	1,200 mg/kg of b.w (for 7 days)	Asala et al. (2021)
*Olea europaea* L	Leaves	Aqueous extract	Rats	Protective effect on CCl_4_-provoked liver toxicity	80 mg/kg of b.w (for 10 days)	Ustuner et al. (2018)
	Leaves	Unspecified	Rats	Protective effect in CCl_4_-caused hepatotoxicity	80 mg/kg of b.w (for 3 days)	Vidičević et al. (2020)
	Fruit pulp	Ethanol, n-hexane, ethyl acetate (EA)extracts	Rats	Hepatoprotective effect against high-fat diet-fed	100–300 mg/kg of b.w (for 28 days)	Kim et al. (2014)
*Piper nigrum* L	Fruits	Methanol extract	Human liver microsomes	Prevent liver	5%	Usia et al. (2005)
	Seeds	hydroalcoholic extract	Mice	Hepatoprotective effects on concanavalin A-provoked liver toxicity	400 mg/kg of b.w	Mushtaq et al. (2021)
	Fruits	Ethanol extract	Rats	Inhibited liver fibrosis induced by thioacetamide	100 mg/kg of b.w (for 28 days)	Dinakar et al. (2010)
	Fruits	Essentiel oils	Mice	Hepatoprotective effect on CCl_4_-induced toxicity in mice	2 g/kg of b.w (for 14 days)	Zhang et al. (2021)
*Pistacia lentiscus* L	Unspecified	Essential oils	Rats	Hepatoprotective effect in rats intoxicated by CCl_4_	2–5 mL/kg of b.w (every 3 days for 15 days)	Maameri et al. (2015)
*Rosmarinus officinalis* L	Leaves	Methanol extract	Rats	Against CCl_4_-provoked liver cirrhosis	200 mg/kg of b.w (for 12 weeks)	Alhussaini (2015b)
	Leaves	Etahanol extarct	Mice	Limits weight gain and liver steatosis in mice fed a high-fat diet	200 mg/kg of b.w (for 50 days)	Harach et al. (2010)
	Leaves	Etahanol extarct	*Oncorhynchus mykiss*	Reducing the rate of steatosis in the liver of rainbow trout	0.4–3 g/kg of b.w (for 40 days)	Farooqui et al. (2016b)
	Leaves	Hydroalcoholic extract	Rats	Hepatoprotective effect of on acetaminophen-caused liver damage	100–500 mg/kg of b.w (for 7 days)	Lucarini et al. (2014)
	Unspecified Unspecified		Rats	Prevent liver against etoposide chemotherapy-caused hepatotoxicity	220 mg/kg of b.w (for 6 weeks)	Almakhatreh et al. (2019)
	Leaves	Hydroalcoholic extract	Rats	Hepatoprotective effect on bile-duct ligation provoked toxicity	500 mg/kg of b.w (for 14 days)	Sadeghi et al. (2020)
	Leaves	Ethanolic Extract	Rats	Prevent liver on alcohol-provoked hepatocytes damage	200 mg/kg of b.w (for 90 days)	Aouad et al. (2021)
	Leaves	Aqueous extract	Rats	Protective effect on trichloroacetate-caused hepatotoxicity	200 mg/kg of b.w (for 2 months)	Abozid and Farid (2018)
	Whole plant	Dichloromethan and methanol extract	HepG2	Regulates metabolism in HepG2 Cells	0 50 μg/mL (for 24 h)	Tu et al. (2013)
*Rumex vesicarius* L	Whole plants	Methanolic extract	Rats	Hepatoprotective effect on CCl_4_-caused toxicity	100–200 mg/kg of b.w (for 7 days)	Ganaie et al. (2015)
	Whole plants	Methanolic extract	Rats	Protective effect in malathion hepatotoxicity	200 mg/kg of b.w (for 28 days)	Mostafa et al. (2018)
*Salvia officinalis* L	Unspecified	Essential oils	Mice	Hepatoprotective effect against high-fat diet exposition	4 mg/kg of b.w (during 8 weeks)	Koubaa-Ghorbel et al. (2020a)
	Unspecified	Essential oils	Rats	Hepatoprotective effects against vanadium-induced oxidative stress and histological changes in the rat liver	15 mg/kg of b.w	Koubaa et al. (2021a)
	Flowers	Aqueous extract	Rats	Hepatoprotective effect against ethanol induced oxidative stress in rats	50–200 mg/kg of b.w (for 15 days)	Jedidi et al. (2022a)
	Leaves	Hydromethanolic extract	Rats	Hepatoprotective effect against *Aspergillus parasiticus* Aflatoxin-caused liver damage in rats	25–150 mg/kg of b.w	Parsai et al. (2015)
	Aerial parts	Hydroalcoholic extract	Rats	Hepatoprotective effect against isoniazid provoked hepatic damage in rats	100–400 mg/kg of b.w (for 28 days)	Shahrzad et al. (2014a)
	Leaves	Ethanolic extract	HepG2	Prevent HepG2 cells against oxidative stress	0.01–100 mg/mL (for 24 h)	Kozics et al. (2013a)
*Syzygium aromaticum* (L.)	Flower buds	Etahanol extarct	Rats	Protective effect on hepatotoxicity caused by thioacetamide	800 mg/kg of b.w (for 3 days)	Prasad et al. (2010)
*Terminalia arjuna* (Roxb. ex DC.) Wight and Arn	Bark	Aqueous extract	Mice	Protect the liver tissues against CCl_4_-caused hepatotoxicity	50 mg/kg of b.w (for 1 week)	Manna et al. (2006)
	Bark	Ethanolic extract	HepG2	Thherapeutic effects on human hepatoma cell line, HepG2, and exhibits its cytotoxicity to these cells, and the cell death is mediated by apoptosis	20–100 mg/L (for 48 h)	Sivalokanathan et al. (2006)
	Bark	Aqueous extract	Rats	Hepatoprotective effect against isoniazid provoked toxicity in rats	200 mg/kg of b.w (for 10 days)	Doorika and Ananthi (2002)
	Fruits	Aqueous and ethanol extracts	Mice	Protective effect on cadmium-caused hepatotoxicity	100 mg/kg of b.w (for 7 days)	Ghosh et al. (2010a)
	Bark	Alcoholic and aqueous extracts	Human liver microsomes	Modulatory impacts on the enzyme activity of CYP3A4, CYP2D6, and CYP2C9 in hepatocyte microsomes	2.5–75 μg/mL	Varghese et al. (2015)
	Bark	Aqueous extract	HepG2	Attenuates toxicity provoked by tert-butyl hydroperoxide in HepG2 cell	25–100 mg/mL	Shivananjappa et al. (2013)
	Bark	Aqueous extract	Rats	Prevent liver against acetaminophen	250–500 mg/kg of b.w (for 14 days)	Kannappan et al. (2020)
	Bark	Aqueous extract	Rats	Protective effect against alcohol caused hepatoxicity in rats	250–500 mg/kg of b.w	Ch et al. (2015)
	Bark	Aqueous extract	HepG2	Reduce basal oxidative stress in HepG2 cells	25–100 μg/mL	Shivananjappa and Joshi (2012)
	Leaves	Aqueous extract	Rats	Attenuated the physiological and histopathological alterations in liver provoked by cisplatin	400 mg/kg of b.w (for 14 days)	Sneha et al. (2021)
	Bark	Aqueous extract	Rats	Protective effects against arsenic-caused aggravation of high fat diet-induced oxidative stress-mediated damages in liver	20–60 mg/kg of b.w (for 8 days)	Dutta et al. (2014)
	Bark	Aqueous extract	Rats	Protective effect against adrenaline-induced hepatic damage in rats through an antioxidant mechanism	10–40 mg/kg of b.w (for 5 days)	Ghosh et al. (2010b)
	Fruits	Ethanolic extaract	Mice	Hepatoprotective effect in acetaminophen intoxicated mice	400 mg/kg of b.w	Saira et al. (2020)
	Bark	Aqueous extract	Rats	Protective effect on acetaminophen-provoked liver damage	250–500 mg/kg of b.w (for 14 days)	Chester et al. (2017)
	Bark	Ethanolic extaract	Rats	Protective effect on paracetamol provoked liver toxicity	200 mg/kg of b.w (once daily for 7 days)	Sangamithira et al. (2011)
	Stem bark	Alcohol extract	Rats	Hepatoprotective effect on CCl_4_-caused toxicity	250–500 mg/kg of b.w (for 5 days)	Anbalagan et al. (2007)
*Thymus vulgaris* L	Unspecified	Alcoholic extract	Broiler chickens	Regulates lipid metabolism in the liver	0.2%–0.6% (for 42 days)	Abdulkarimi et al. (2011)
	Unspecified	Essential oil	Rats	Enhances the total antioxidant potential of hepatocytes	42.5 mg/kg of b.w	Youdim and Deans (1999)
	Leaves	Unspecified	Mice	Boost the activity of xenobiotic-metabolizing enzymes in the liver	0.5%–2.0% (for 7 days)	Sasaki et al. (2005)
	Unspecified	Essential oils	Japanese quails (*Coturnix coturnix* japonica)	Ameliore *in vivo* antioxidant activity in the liver	150–450 mg/kg of b.w	Nutrition et al. (2017)
	Leaves, flowers and stems	Aqueous extract	Rats	Hepatoprotective effect against toxicity in rats exposed to aluminum	150 mg/kg of b.w (daily for 90 days)	Mokrane et al. (2020)
	Unspecified	Aqueous extract	Rats	Improved liver injury induced by paclitaxel in rats	4.5–18 mg/kg of b.w (for 2 weeks)	Salahshoor et al. (2019)
	Aerial parts	Aqueous extract	Rats	Prevent liver against dexamethasone-provoked liver damage	500 mg/kg of b.w (for 8 weeks)	Abou-Seif et al. (2019)
	Leaves	Ethanolic extaract	HepG2	Protective the HepG2 cells oxidative stress	100 mg/mL	Kozics et al. (2013b)
*Ziziphus lotus* (L.) Lam	Fruits	Aqueous extract	Rats	Protective effect on CCl_4_ provoked liver toxicity	200 and 400 mg/kg for 14 days	Bencheikh et al. (2019)

According to the results of our ethnobotanical survey, *C. cyminum* L. (UV = 0.1065), *A. sativum* L. (UV = 0.1015), *S. officinalis* L. (UV = 0.0761), *Asparagus officinalis* L. (UV = 0.0558), and *Z. lotus* (L.) Lam. (UV = 0.0457) are the medical species commonly used in Northeastern Morocco for the treatment or prevention of liver problems. To support the use of these plants in conventional medicine, it may be interesting to further explore and discuss their pharmacological properties related to liver problems. To this goal, we shall explore the pharmacological potential of these herbs in the following paragraphs to validate their benefits against liver diseases:

*Allium sativum* L. is ranked as the second most used species (UV = 0.1015) for the treatment of liver diseases. According to ethnobotanical findings, this plant is widely used in rural areas of northeastern Morocco for its hepatoprotective effect, against liver cancer, and viral infections (hepatitis). The leaves and bulb of *A. sativum* have demonstrated antioxidant and hepatoprotective effects against ethanol-induced hepatotoxicity in rats (Nencini et al., 2010). Indeed, the administration of an extract of the leaves or bulbs of *A. sativum* At a dose of 250 mg/kg, the Glutathion reductase (GR), catalase (CAT), and superoxide dismutase (SOD) activities were restored, and the levels of malondialdehyde, ascorbic acid, and glutathion were reduced and oxidized in the liver tissue of rats exposed to ethanol (Nencini et al., 2010). A study also discovered that *A. sativum* has a cytoprotective impact in HepG2 cells submitted to mycotoxines, specifically Beauvericin, α-Zearalenol and β-Zearalenol (Juan-García et al., 2021b). The presence of antioxidant compounds, according to the authors, is responsible for this cytoprotective effect, which involves the activation of defensive pathways as an enzymatic defence mechanism from within cells, the control of the cell cycle, and cell death, all of which can be provoked by these mycotoxines (Juan-García et al., 2021b). Another research revealed that an aqueous extract of garlic bulbs reduces alloxane elevation of biological parameters of liver and kidney functions in rats (Aprioku and Amah-Tariah, 2017b). According to prior study, garlic includes a number of bioactive components such as organosulfur compounds, saponins, and phenolic compounds (Bradley et al., 2016; Diretto et al., 2017). Organosulfur compounds such as diallyl thiosulfonate (allicin), diallyl sulfide, diallyl disulfide, diallyl trisulfide, E/Z-ajoene, S-allyl-cysteine, and S-allyl-cysteine sulfoxide have been claimed to be the principal active phytochemicals found in garlic (Figure 5) (Yoo D. Y. et al., 2014; Yoo et al., 2014 M.; Kodera et al., 2017; Mansingh et al., 2018). These compounds were discovered to be related to the plant’s powerful antioxidant and antitumor ability (Bagul et al., 2015).

*Asparagus officinalis* L. this plant is ranked third among the plants most commonly used for the treatment of liver diseases by the Moroccan population. It is frequently used to treat biliary problems, hepatic stones, hepatitis, and jaundice, as shown in Table 3. It has been reported that the aqueous and ethanolic extract of *A. officinalis* have hypolipidemic and hepatoprotective effects in mice fed a high-fat diet (Zhu et al., 2010). According to the findings of this study, daily treatment of 200 mg/kg of either ethanolic or aqueous extract for 8 weeks enhanced lipid parameters, transaminase (Alanine and Aspartate) activity, superoxide dismutase (SOD) and antioxidant capacity, and hepatic malondialdehyde levels. In addition, an *in vivo* research indicate that the aqueous extract of *A. officinalis* roots has protective properties on cadmium chloride-induced liver injury in rats (Abedi et al., 2018). This investigation found that treatment by aqueous extract of *A. officinalis* roots at 200 and 400 mg/kg for 28 days significantly restored liver biomarkers in cadmium chloride poisoned rats. Several phytochemical investigations have revealed that the main bioactive compounds found in asparagus include phenolic compounds, sterols, and saponins (Jang et al., 2004; Fuentes-Alventosa et al., 2013). Asparanin A, Asparoffin C, Asparoffin D, Asparenyol, Gobicusin B, Protodioscin and 1-methoxy-2-hydroxy-4-[5-(4-hydroxyphenoxy)-3-penten1-ynyl] phenol are the main phytochemicals found in asparagus, with Rutin (Quercetin 3-rutinoside) as the major compound (Figure 5) (Fan et al., 2015). These compounds’ antioxidant action is well-known (Sun et al., 2007; Solana et al., 2015; Slatnar et al., 2018), that may contribute in the hepatoprotective effects of the plant.

**FIGURE 6:**
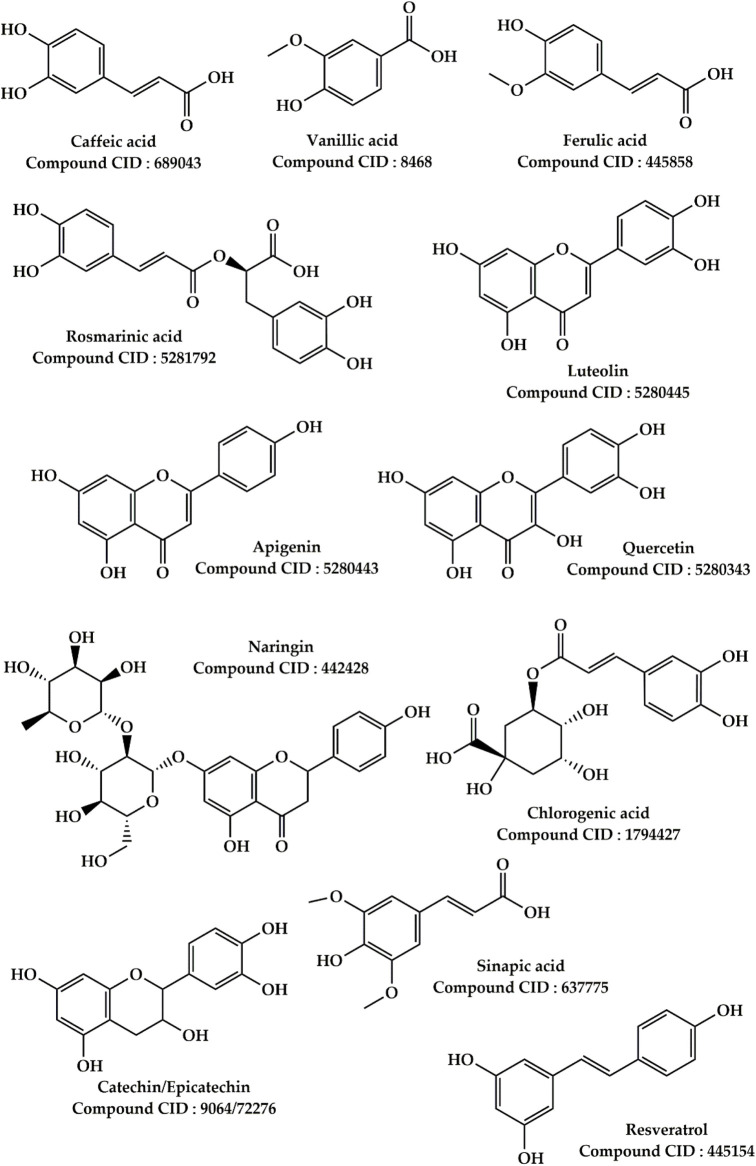
Main phytoconstituents found in *Salvia officinalis* L. and *Ziziphus lotus* (L.) Lam.

*Salvia officinalis* L. This plant is widely used in folk medicine in North East Morocco to treat liver failure. It is ranked fourth of the most commonly used plants, with a use value of 0.0761. According to our findings, various parts of this plant have traditionally been used to treat jaundice, hepatitis, and liver cancer in the study site. Several preclinical investigations on plant parts were carried out to examine its medicinal qualities for liver failure. In fact, 8 weeks of daily administration of *S. officinalis* essential oil at 4 mg/kg enhanced hyperlipidemia, hepatic, and renal lesions in mice fed a high-fat diet (Koubaa-Ghorbel et al., 2020b). This effect of *S. officinalis* essential oil was more effective than that of simvastatin (standard drug for this purpose). In addition, daily intake of 15 mg/kg of the essential oil of *S. officinalis* showed a protective effect against vanadium-induced hepatotoxicity in Wistar rats (Koubaa et al., 2021b). The treatment of rats with 200 mg/kg of *S. officinalis* aqueous extract for 15 days showed protective effects against ethanol-induced hepatotoxicity (Jedidi et al., 2022b). According to the same authors, this effect is reflected in the improvement of plasma transaminase activity and the restoration of hepatocyte structure in rats poisoned with ethanol. Besides, it was shown that administering a hydroalcoholic extract of *S. officinalis* at a dose of 250 mg/kg protected rats from isoniazid-induced hepatotoxicity (Shahrzad et al., 2014b). Furthermore, a previous investigation showed that an ethanolic extract of *S. officinalis* leaves protects human HepG2 cells from hydrogen peroxide and 2,3-dimethoxy-1,4-naphthoquinone-induced DNA damage (Kozics et al., 2013b). As shown in Figure 6, common sage contains a variety of biologically active compounds, primarily two types of relatively abundant phenolic components: phenolic acids (caffeic, vanillic, ferulic, and rosmarinic acids) and flavonoids (luteolin, apigenin, and quercetin) (Lu and Foo, 2002; Roby et al., 2013). These phenolic components are well-known as hepatoprotective agents (Kiokias and Oreopoulou, 2021; Venmathi Maran et al., 2022).

*Ziziphus lotus* (L.) Lam. According to our ethnobotanical study conducted in several parts of the Moroccan North-East, this plant ranks sixth among the most commonly utilized herbs to cure liver disorders. Indeed, the leaves and fruits of this plant were utilized to treat jaundice, hepatic colic, and hepatitis in the research region. Previous pharmacological work has demonstrated that *Z. lotus* extracts exert hepatoprotective effects at the preclinical stage. In a rat investigation, an aqueous extract of *Z. lotus* fruits was found to have hepatoprotective properties against CCl_4_-induced liver damage (Bencheikh et al., 2019). The findings of this study indicate that administration of aqueous extract of *Z. lotus* fruits at doses of 200 and 400 mg/kg restored the biochemical parameters (liver biomarkers) altered during hepatotoxicity induced by CCl_4_ injections in rats. Similarly, it has been reported that treatment of rats with the aqueous extract of *Z. lotus* fruit at doses of 200 and 400 mg/kg protects the liver and kidney from gentamicin poisoning (Bencheikh et al., 2021b). In the literature, it has been well demonstrated that the hepatotoxicity caused by the agent CCl_4_ and gentamicin is related to the oxidative stress caused by these chemical compounds (Lin and Huang, 2000; Achuthan et al., 2003). In this context, several authors confirm that the use of natural antioxidants to fight against the oxidative stress caused by CCl_4_ and gentamicin is the best strategy to prevent hepatotoxicity produced by these hepatotoxic substances (Bencheikh et al., 2021a; Bouhrim et al., 2021; Ouahhoud et al., 2021b). Extracts of *Z. lotus* fruits are high in phenolic compounds such as Rutin, Naringin, Chlorogenic acid, Rosmarinic acid, Quercetin, Cat-echin, Epicatechin, Sinapic acid, Resveratrol, and Caffeic acid, according to phytochemical research (Figure 6) (Marmouzi et al., 2019; Bencheikh et al., 2021c; 2021d). These photochemical compounds thanks to their antioxidant powers could be responsible for the hypatoprotective effects.

## 4 Conclusion

This ethnobotanical study reveals that locals in remote areas of northern Morocco possess extensive traditional knowledge about using medicinal plants to treat liver diseases, reflecting the region’s floristic richness. The findings demonstrate the potential of these herbs in addressing liver-related health issues within these communities. However, caution is necessary when using these remedies. The study is limited by its small sample size and lack of a control group, which may affect the robustness of the conclusions.

Further research is essential to evaluate the pharmacological benefits and phytochemical components of these plants, identify active ingredients, and confirm their clinical efficacy. Additionally, safety data are needed to standardize dosages and ensure safe use. Addressing these limitations will help in the development of effective medications derived from these medicinal plants for liver disease treatment.

## Data Availability

The original contributions presented in the study are included in the article/supplementary material, further inquiries can be directed to the corresponding authors.
