# Corrigendum: Ethnobotanical survey and scientific validation of liver-healing plants in northeastern Morocco

**DOI:** 10.3389/fphar.2024.1508059

**Published:** 2024-12-03

**Authors:** Noureddine Bencheikh, Amine Elbouzidi, Abdellah Baraich, Mohamed Bouhrim, Abdelhamid Azeroual, Mohamed Addi, Ramzi A. Mothana, Hanan M. Al-Yousef, Bruno Eto, Mostafa Elachouri

**Affiliations:** ^1^ Agri-Food and Health Laboratory (AFHL), École Supérieure Normale, Hassan First University, Settat, Morocco; ^2^ Laboratoire d’Amélioration des Productions Agricoles, Biotechnologie et Environnement (LAPABE), Faculté des Sciences, Université Mohammed Premier, Oujda, Morocco; ^3^ Laboratory of Bioressources, Biotechnology, Ethnopharmacology and Health, Faculty of Sciences, Mohammed First University, Oujda, Morocco; ^4^ Laboratory of Biological Engineering, Team of Functional and Pathological Biology, University Sultan Moulay Slimane Faculty of Sciences and Technology Beni Mellal, Meknes, Morocco; ^5^ Department of Pharmacognosy, College of Pharmacy, King Saud University, Riyadh, Saudi Arabia; ^6^ Laboratories TBC, Laboratory of Pharmacology, Pharmacokinetics and Clinical Pharmacy, Faculty of Pharmacy, University of Lille, Lille, France

**Keywords:** ethnobotany, ethnopharmacology, traditional medicine, medicinal plants, liver diseases

In the published article, there was an error regarding the **Affiliation** of Mohamed Bouhrim. Instead of having both “Laboratory of Biological Engineering, Team of Functional and Pathological Biology, University Sultan Moulay Slimane Faculty of Sciences and Technology Beni Mellal, Meknes, Morocco” and “Department of Pharmacognosy, College of Pharmacy, King Saud University, Riyadh, Saudi Arabia,” they should only be affiliated with **Affiliation 4**, “Laboratory of Biological Engineering, Team of Functional and Pathological Biology, University Sultan Moulay Slimane Faculty of Sciences and Technology Beni Mellal, Meknes, Morocco.”

The authors apologize for this error and state that this does not change the scientific conclusions of the article in any way. The original article has been updated.

